# The correction of conjunctivochalasis using high-frequency radiowave electrosurgery improves dry eye disease

**DOI:** 10.1038/s41598-021-82088-5

**Published:** 2021-01-28

**Authors:** Yong Woo Ji, Hyojin Seong, Sujung Lee, Mutlaq Hamad Alotaibi, Tae-im Kim, Hyung Keun Lee, Kyoung Yul Seo

**Affiliations:** 1 Department of Ophthalmology, National Health Insurance Service Ilsan Hospital, Goyang, South Korea; 2 Department of Ophthalmology, Institute of Vision Research, Yonsei University College of Medicine, 50-1 Yonsei-ro, Seodaemun-gu, Seoul, 03722 South Korea; 3 Medical Library, National Health Insurance Service Ilsan Hospital, Goyang, South Korea; 4 Department of Ophthalmology, Corneal Dystrophy Research Institute, Yonsei University College of Medicine, Seoul, South Korea; 5 Department of Ophthalmology, Prince Mohammad Bin Abdulaziz Hospital, Riyadh, Saudi Arabia

**Keywords:** Conjunctival diseases, Corneal diseases, Outcomes research, Eye manifestations

## Abstract

We aimed to determine the clinical impact of conjunctivochalasis (CCh) and its correction using high-frequency radiowave electrosurgery (HFR-ES), for signs and symptoms of dry eye disease (DED). Forty patients diagnosed with symptomatic CCh were prospectively enrolled. As a result, patients with CCh had moderate to severe DED and most of them exhibited meibomian gland dysfunction (MGD). Corneo-conjunctival fluorescein staining score (CFS) and all lid-parallel-conjunctival-folds scores (LIPCOFs) were positively correlated. Nasal LIPCOF significantly correlated with symptoms and tear volume. Central, temporal, and total LIPCOF significantly correlated with MG loss, MGD stage, and lipid layer thickness. Independent significant factors associated with total LIPCOF included CFS, tear break-up time, and MGD stage. One month following HFR-ES, CCh was completely resolved in all cases. Patient age and preoperative nasal LIPCOF were determinants of outcomes associated with postoperative improvements in symptoms. Ocular surface parameters significantly improved, but MGD-related signs did not. Collectively, CCh associated with MGD severity deteriorates not only tear film stability and reservoir capacity, leading to DED exacerbation. Therefore, CCh should be corrected in patients with DED and MGD. Younger patients with nasal CCh are likely to experience more symptomatic relief after HFR-ES. Particularly, management for MGD should be maintained after CCh correction.

## Introduction

Conjunctivochalasis (CCh) is a chronic condition of the ocular surface (OS), and its prevalence increases with age^1,2^. CCh is most common in elderly individuals and is one of the most common co-morbid conditions in patients with dry eye disease (DED)^3–10^. CCh comprises redundant conjunctival folds characterized by loose and non-edematous bulbar conjunctiva that accumulate between the eyeball and the eyelid margin^2,11^. CCh is often found between the globe and lower eyelid, but in severe cases can be observed in the superior and even the whole bulbar conjunctiva^2,12^. CCh has been thought to be caused by elastic fiber degradation involving inflammatory factors or mechanical interference with conjunctival lymphatic circulation^13–15^. Recently, CCh is considered to be one of the friction-related diseases (FRD), which also include superior limbic keratoconjunctivitis and lid wiper epitheliopathy^16^.

Previous studies demonstrated that the presence of CCh exhibits a strong positive predictive value in the diagnosis of DED^8,10^. Furthermore, CCh can symptomatically mimic DED, since it negatively affects the stability, distribution, and clearance of tear film^2,4,6,17,18^. The mechanistic relationship of CCh to DED severity depends on the friction induced by blinking and low tear volume^7,16,19^. Moreover, DED signs and symptoms are more severe when meibomian gland dysfunction (MGD) coexists with FRDs such as CCh^5,16^.

CCh causes not only epiphora and ocular discomfort, but also adversely impacts vision-related quality of life due to the highly unstable tear film^20^. Accordingly, symptomatic CCh should be treated following an attentive diagnosis. Clinicians have used anterior segment optical computed tomography (AS-OCT) as well as routine slit-lamp biomicroscopy to inspect conjunctival folds^3,10,12^. The first-line treatment for CCh is medical therapy, including use of OS lubricant and anti-inflammatory eyedrops^21^. However, in advanced cases, to alleviate ocular signs and symptoms of DED, surgical approaches such as excision, cauterization, or high-frequency radiowave electrosurgery (HFR-ES) should be considered^12,21–25^.

**Figure 1:**
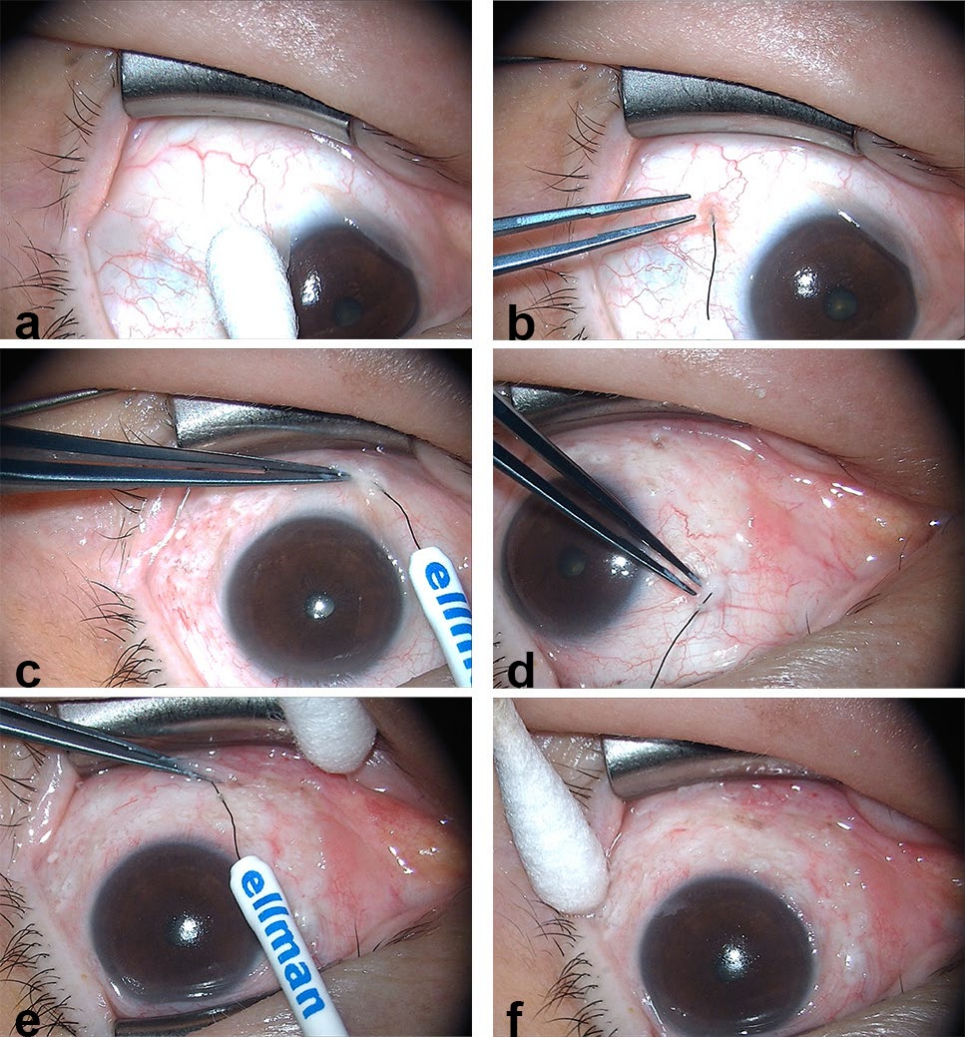
Consecutive photographs of high-frequency radiowave electrosurgery for conjunctivochalasis. (**a**) After instillation of 0.5% proparacaine, the extent of the conjunctivochalasis (CCh) was determined using a cotton tip. (**b**–**d**) Using a fine insulated coated needle of the 4.0-MHz radiowave system, ablation of CCh was consecutively performed at the temporal, inferior, and nasal bulbar conjunctiva, causing a gradual change of the conjunctiva to a dusky white color (blanching) while lifting to prevent the sclera from thermal burns. (**e**) Ablation was also continued at the conjunctival fornix. (**f**) Finally, complete elimination of CCh was confirmed using a cotton tip.

In this study, we aimed to investigate the clinical impact of CCh and its correction using HFR-ES (Fig. 1), with regard to the signs and symptoms of DED. Furthermore, to provide information for proper management after surgery, we determined the factors associated with the CCh severity and symptom relief following HFR-ES.

## Results

Forty eyes (40 patients) with symptomatic CCh were prospectively enrolled. The mean age of patients was 61.65 ± 12.39 years, and 40% were women. lid-parallel conjunctival folds (LIPCOF) scores were representative of CCh (nasal, 2.28 ± 0.64; central, 0.85 ± 0.98; temporal, 2.10 ± 0.81; total, 5.23 ± 1.82). Table 1 shows that patients enrolled in the present study had moderate to severe DED (OS Disease Index (OSDI), 42.65 ± 19.88; tear film break-up time (TBUT), 1.83 ± .55 s; corneo-conjunctival fluorescein staining score (CFS), 1.43 ± .50) and 82.5% of them had MGD (MGD stage, 1.60 ± 1.53; meibomian gland loss (MGL) grade, 1.45 ± 1.36). Moreover, tear meniscus cross-sectional areas (TMAs) at all regions of the lower lid margin were low (nasal,.0045 ± .0051 mm^2^; central, .0103 ± .0086 mm^2^; temporal, .0061 ± .0059 mm^2^) (Table 1).

**Table 1:** Demographic characteristics of patients enrolled in the present study.

	n = 40
Age, years (range)	61.65 ± 12.39 (34–82)
Sex, n (%), female	16 (40)
OSDI (range)	42.65 ± 19.88 (20.45–72.73)
TBUT, s (range)	1.83 ± 0.55 (1–3)
CFS score (range)	1.43 ± 0.50 (1–2)
**LIPCOF score (range)**	
Nasal	2.28 ± 0.64 (1–3)
Central	0.85 ± 0.98 (0–3)
Temporal	2.10 ± 0.81 (1–3)
Total	5.23 ± 1.82 (3–9)
**TMA, mm**^**2**^** (range)**	
Nasal	0.0045 ± 0.0051 (0–0.015)
Central	0.0103 ± 0.0086 (0–0.024)
Temporal	0.0061 ± 0.0059 (0–0.015)
LLT, nm (range)	72.43 ± 27.44 (21–100)
MGD stage (range)	1.60 ± 1.53 (0–4)
MGL grade (range)	1.45 ± 1.36 (0–4)

All data are shown as the mean ± standard deviation. OSDI, ocular surface disease index; TBUT, Tear break-up time.

CFS, corneal and conjunctival fluorescein staining based on Oxford schema; LIPCOF, Lid-parallel conjunctival folds; TMA, tear meniscus cross-sectional area; LLT, Lipid layer thickness; MGD, Meibomian gland dysfunction; MGL, Meibomian gland loss.

### Correlation between CCh and ocular surface parameters and investigation of clinical determinants associated with the severity of CCh

**Figure 2:**
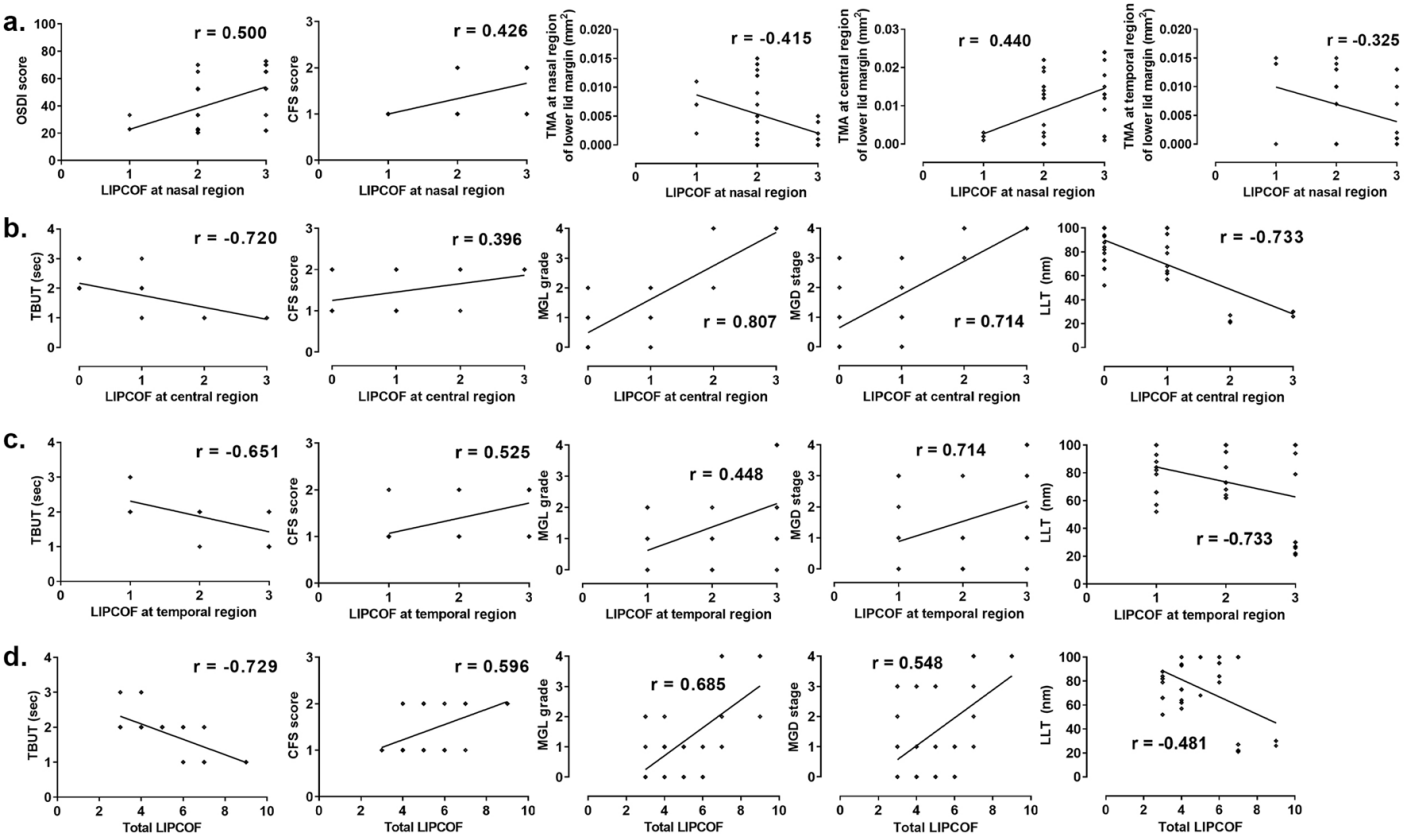
Correlation between the severity of conjunctivochalasis and ocular surface parameters. Among all ocular surface indexes, significant parameters related to each conjunctivochalasis (CCh) are shown with their coefficients (*r*) after the Spearman correlation analysis. (LIPCOF, lid-parallel conjunctival folds; OSDI, ocular surface disease index; CFS, corneoconjunctival fluorescein staining based on Oxford schema; TMA, tear film meniscus cross-sectional area measured by anterior segment optical computed tomography; TBUT, tear film break-up time; MGL, meibomian gland loss; MGD, meibomian gland dysfunction; LLT, lipid layer thickness).

We first investigated which ocular surface parameters had pathological relationships to CCh. Correlation analysis revealed a strong positive correlation between nasal LIPCOF and OSDI score (*r* = .500 and *P* = .001, Fig. 2a). In addition, nasal LIPCOF score negatively correlated with nasal and temporal TMAs (*r* =  − .415, − .325 and *P* = .004, .020, respectively), but positively correlated with central TMA (*r* = .440 and *P* = .002, Fig. 2a). Nasal LIPCOF was not significantly correlated with TBUT, while central, temporal, and total LIPCOF were significantly correlated (*r* =  − .151, − .720, − .651, 729, and *P* = .176, < .001, < .001, < .001, respectively, Fig. 2b–d). Central and temporal as well as total LIPCOF were significantly related to MGD-related factors, including MGL grade, MGD stage, and lipid layer thickness (LLT) (Fig. 2b–d). There was a positive correlation between CFS and all LIPCOFs (Fig. 2).

Next, we explored which clinical determinants were associated with CCh severity. Since all clinical signs or measurements for DED and MGD reflected the whole area of OS or MG, we analyzed factors associated with total LIPCOF score using multiple mixed linear regression. Interestingly, multivariable analysis revealed that significant independent factors associated with total LIPCOF included CFS, TBUT, and MGD stage (β = .416, − .434, .222 and *P* =  < .001, .001, < .046, respectively). However, our analysis showed MGL grade and LLT did not affect total LIPCOF (*P* = .962 and .326, respectively), inconsistent with the correlation analysis displayed in Fig. 2 (Table 2).

**Table 2:** Clinical parameters influencing severity of conjunctivochalasis in patients with dry eye disease**.**

Dependent variable	Explanatory variables	Correlation analysis		Multivariable analysis				
				Unstandardized coefficients		Standardized coefficients	*P*-value	Adjusted R^2^
		*r*	*P*-value	B	SE	β		
LIPCOF score—total	**CFS score**	**.596**	**< .001**	**1.510**	**.335**	**.416**	**< .001**	**.719**
	**TBUT**	**− .729**	**< .001**	**− 1.437**	**.375**	**− .434**	**.001**	
	**MGD stage**	**.685**	**< .001**	**0.263**	**.127**	**.222**	**.046**	
	MGL grade	.548	< .001			.010	.962	
	LLT	− .481	.001			.132	.326	

Significant values are presented in bold type. Each *P-*value was obtained via Spearman correlation analysis or stepwise multivariable regression analysis; *r,* Spearman’s correlation coefficient.

SE, standard error; LIPCOF, Lid-parallel conjunctival folds; CFS, corneal and conjunctival fluorescein staining based on Oxford schema; TBUT, tear break-up time; MGD, Meibomian gland dysfunction; MGL, Meibomian gland loss; LLT, Lipid layer thickness.

### Effect of CCh correction on ocular surface signs and symptoms

**Figure 3:**
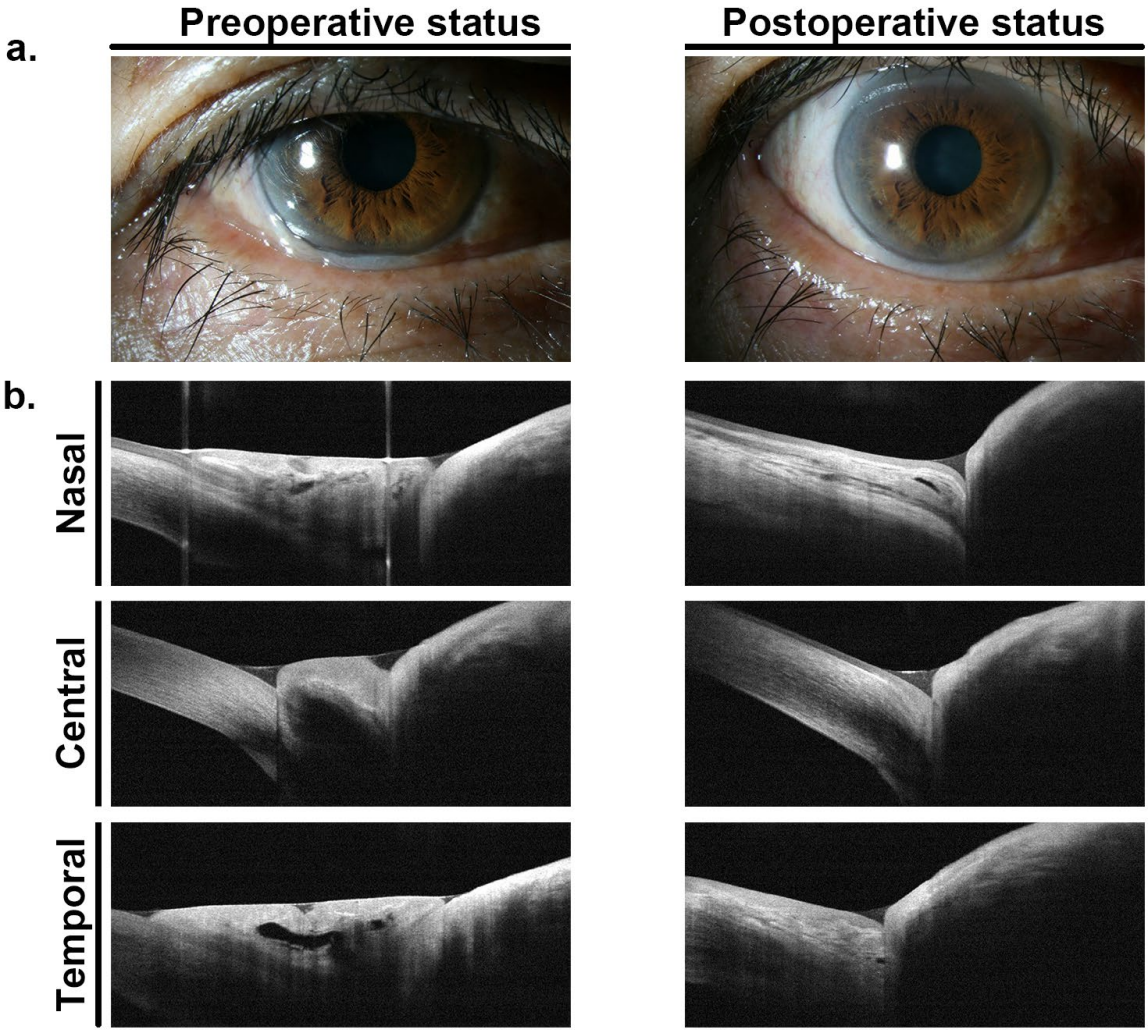
Representative photographs of the surgical effect of high frequency radiowave electrosurgery for conjunctivochalasis. (**a**). At one month after high-frequency radiowave electrosurgery for conjunctivochalasis (CCh), complete elimination of CCh was confirmed using slit-lamp biomicroscopy. (**b**) Anterior segment optical computed tomography also identified recovery of tear reservoirs at the nasal, central, and temporal regions of the lower lid margin, as well as removal of redundant conjunctival tissue.

To ameliorate the OS environment disturbed by CCh, we performed HFR-ES for symptomatic CCh patients according to our previous method (Fig. 1)^24,25^. All surgeries were uneventful, and no significant postoperative complication was noted. At one month after HFR-ES, we confirmed complete elimination of CCh in all cases using slit-lamp biomicroscopic examination (Fig. 3a). AS-OCT also identified recovery of tear reservoirs at the nasal, central, and temporal regions of the lower lid margin as well as removal of redundant conjunctival tissues (Fig. 3b).

**Figure 4:**
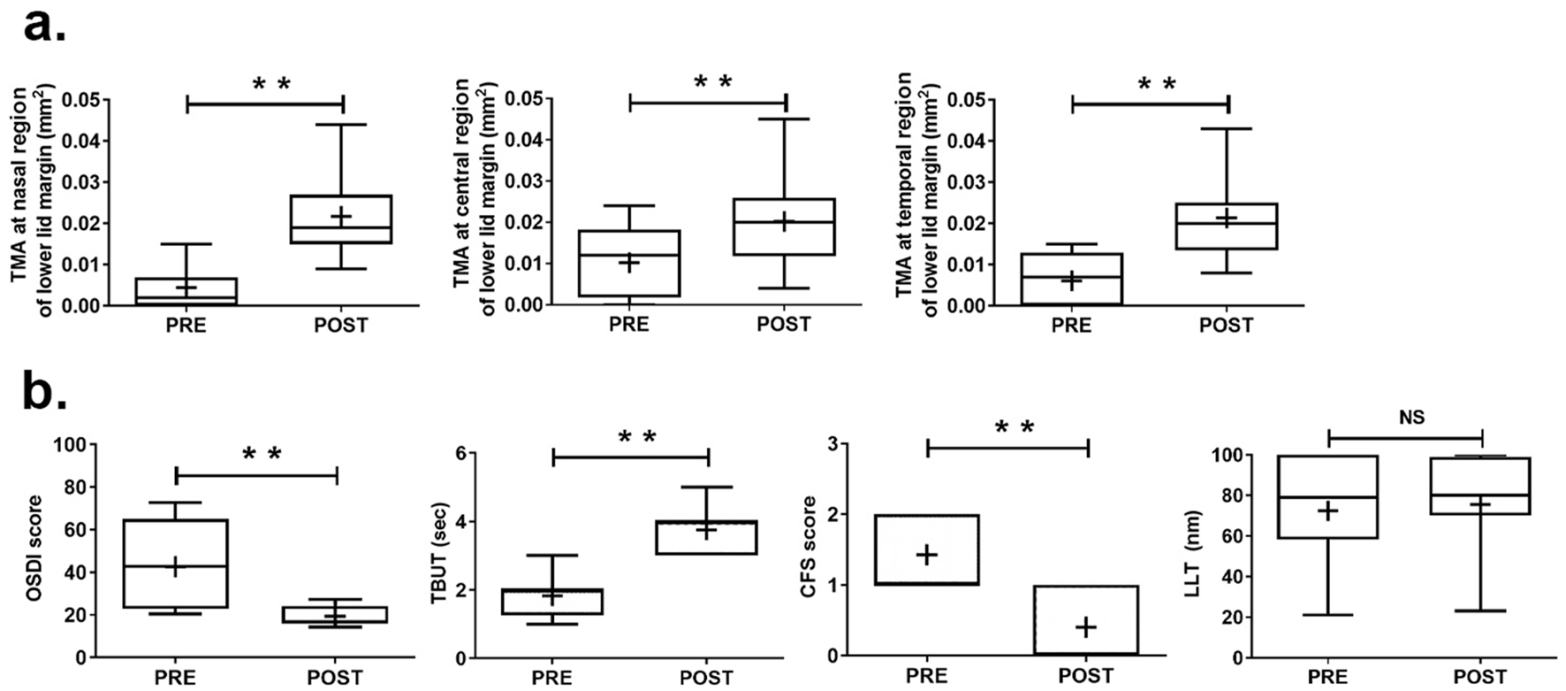
Changes of ocular surface parameters following high-frequency radiowave electrosurgery for conjunctivochalasis. One month after high-frequency radiowave electrosurgery was performed for conjunctivochalasis, ocular surface indexes were compared between the preoperative and postoperative conditions using the Wilcoxon signed-rank test. Among all indexes, meaningful parameters are shown with their statistical significance. (**a**) Tear film meniscus cross-sectional areas measured by anterior segment optical computed tomography (TMA). (**b**) Ocular surface disease index (OSDI), tear film break-up time (TBUT), corneoconjunctival fluorescein staining based on Oxford schema (CFS), and lipid layer thickness (LLT). (**, *P* < .01; NS, not significant).

Consistent with the findings on physical inspection, postoperative TMAs at all regions were significantly increased compared with the preoperative condition (*P* < .01, Fig. 4a; Supplemental Table 1). In addition, there were about two-fold improvements in not only the TBUT and CFS scores but also in the OSDI after surgical treatment for CCh (Fig. 4b, Supplemental Table 1). According to our multivariable analysis, postoperative symptomatic relief scored by OSDI was independently determined by patients’ age and preoperative nasal LIPCOF (β =  − .335,.529 and *P* = .010, < .001, respectively) (Table 3). However, there were no changes in the MGD stage and MGL grade between the pre- and postoperative statuses, and LLT was not also significantly increased after HFR-ES (72.43 ± 27.44 vs. 75.53 ± 26.58 nm) (Fig. 4b, Supplemental Table 1). These results were also confirmed using multivariable analysis, as shown in Table 3.

**Table 3:** Multivariable regression analysis evaluating the effect of clinical variables on symptomatic improvement in dry eye patients following conjunctivochalasis correction.

Dependent variable	Explanatory variables	Correlation analysis		Multivariable analysis				
				Unstandardized coefficients		Standardized coefficients	*P*-value	Adjusted R^2^
		*r*	*P*-value	B	SE	β		
Δ OSDI	**Age**	**− .364**	**.011**	**− .454**	**.165**	**− .335**	**.009**	**.427**
	**Preop. LIPCOF score—nasal**	**.478**	**.001**	**13.860**	**3.257**	**.529**	**< .001**	
	ΔTMA—temporal	.451	.002			.178	.197	
	ΔCFS score	.289	.035			.209	.088	
	Δ LLT	− .421	.003			− .106	.463	

Significant values are presented in bold type. Each *P-*value was obtained via Spearman correlation analysis or stepwise multivariable regression analysis; *r*, Spearman’s correlation coefficient; SE, standard error; Δ, difference between postoperative and preoperative values.

OSDI, ocular surface disease index; LIPCOF, Lid-parallel conjunctival folds; TMA, tear meniscus cross-sectional area; CFS, corneal and conjunctival fluorescein staining based on Oxford schema; LLT, Lipid layer thickness.

## Discussion

In this study, we demonstrated that patients with CCh showed moderate to severe DED and most of them exhibited MGD. Particularly, OS disruption, tear instability, and MGD severity might be contributing factors to the exacerbation of CCh. HFR-ES for CCh was an effective modality for the anatomical correction of OS, and its effect on symptom relief was related to patient age and improvement of CCh in the nasal region. Moreover, it significantly ameliorated OS symptoms and signs, including OS disruption and tear film instability. However, MGD-related factors were not altered, even after CCh correction.

The exact pathological mechanism of CCh remains unclear, but several alterations of OS have been postulated. The first alteration is the senile degradation of the subconjunctival connective tissue, reducing attachment of the bulbar conjunctiva to the sclera and conjunctival laxity^2,14^. A recent transcriptome analysis of human conjunctiva with CCh demonstrated that reduced gene expression could cause decreased cell viability, which is related to the aging process^26^. The second is OS inflammation, which induces elastic fiber breakdown by destructive fibroblasts and infiltrated cells, resulting in initiating or accelerating CCh^13,14,27–29^. Proinflammatory proteins also have been detected in tears of CCh patients^15,30–32^. The third is oxidative stress including lipid peroxidation and DNA damage in the chalatic conjunctiva, causing silent inflammation without recruitment of inflammatory cells^33^. This can contribute to initiation of NF-κB and/or TNF-α signaling in the conjunctiva which can release cytokines in tears. Also, it can induce an increase in collagenolytic activity of conjunctival epithelial and stromal cells. Lastly, mechanical friction of the eyelids and completeness of the blink are also causative^7,16,34^. Recently, researchers have categorized CCh into FRD as the one of DED subtypes^16,35^. In addition, Pult et al*.* revealed that frequent partial blinks are significantly related to LIPCOF grade^7^.

The association between CCh and DED is widely accepted^2^. In the current work, patients with CCh displayed a co-existence of moderate to severe DED. Furthermore, CFS as OS disruption in DED was significantly correlated with LIPCOF grades in all regions. This is consistent with another finding wherein CFS was a determinant of total CCh severity. Particularly, only nasal LIPCOF was significantly related to OSDI in our study. These results are supported by the findings of several previous papers^6,17,36^ which suggested nasally located CCh was associated with higher DED symptoms. Specifically, Wang et al*.* demonstrated that symptoms as well as inflammation of tears and loss of goblet cells were more severe in patients with nasal CCh compared with eyes without nasal CCh^36^. A positive relationship between CCh severity and DED symptoms has been described^9,20,37^. In addition, a multicenter comparative study by Nemeth et al*.* also showed that the LIPCOF grade significantly correlated with subjective and objective signs of DED, even in various DED subgroups^8^. Moreover, a recent population-based study proposed that CCh may be an independent risk factor for DED (odds ratio, 2.57)^5^.

The present findings showed that reduced TBUT was correlated with increase in central and temporal LIPCOFs. CCh causes obliteration of the lower tear reservoir, imitating OS states like aqueous tear deficiency (ATD). Previous study demonstrated that TBUT in ATD combined with FRD like as CCh, was shorter than that of ATD patients without FRD regardless of whether MGD was comorbid^16^. Furthermore, our multivariable analysis also indicated that shorter TBUT contributed to increase in CCh grade. Some studies have illustrated a decrease in soluble and membranous mucin, which affects TBUT, is associated with the severity of CCh^26,38^. On the other hand, there was no relationship between TBUT and nasal LIPCOF but nasal LIPCOF was related to an increase in central TMA. This can be explained by delayed tear clearance with inferior punctal occlusion caused by nasal CCh^2,4^.

Furthermore, we demonstrated that higher MGL grade, MGD stage, and lower LLT were significantly associated with severe CCh in all but the nasal region. However, among our MG-related parameters, only MGD stage independently predicted LIPCOF severity, whereas any measure of tear volume such as TMA was not correlated. Di Pascuale et al*.* reported that spatial correlation of CCh with anterior migration of the mucocutaneous junction was a feature of MGD^34^. Regarding alterations of the location of Marx’s line, Pflugfelder et al*.* proposed that an anterior tear meniscus with concentrated osmolarity and inflammatory cytokines could affect differentiation of the MG orifices, causing squamous metaplasia of the lid margin^39^.

In addition, our results showed a negative correlation between LLT and LIPCOF grade, even though the preoperative LLT in our cohort was relatively high (72.4 nm) compared to that in a previous report (49.6 nm)^40^. Because the LipiView II measures LLT at the lower cornea, LLT in CCh cases may be overestimated due to inferiorly stacking lipids, which in turn may be caused by inadequate spread of the tear layer. Insufficient meibum distribution into tears may lead to lipid tear deficiency (LTD), resulting in friction-related microtrauma and inflammation on the OS. In fact, in 1988, Meller and Tseng suggested that an unstable precorneal tear film in CCh patients might be caused by LTD rather than ATD^4^. Collectively, it is probable that LTD with tear instability and OS disruption causes symptomatic CCh.

To date, it is well acknowledged that CCh surgical reconstruction of the tear meniscus as well as the tear reservoir can improve ocular symptoms and signs, including TBUT, tear osmolarity, inflammatory markers (i.e. matrix metalloproteinase-9), and contrast sensitivity^15,19,22,23,40^. In the current study, there were consistent and significant improvements in OSDI, TBUT, and CFS even at one month after HFR-ES. Furthermore, this electrosurgical approach may lead to significant recovery of TMAs at all regions. Consistent with our findings, Qiu et al*.* also reported that TMA increased significantly three months after use of the conjunctival excision method^23^.

According to other studies, postoperative Schirmer’s test results did not significantly change despite an improvement in patient symptoms^23,40^. The authors of these studies suggested that ATD was not strongly associated with symptoms in patients with CCh. Our data also showed changes in TMA were not independently associated with symptomatic improvements. Furthermore, there was no significant change in the MGL grade, LLT, and MGD stage between the pre- and post-operative statuses, indicating that LTD did not appear to be a major contributor to CCh symptoms. In support of this, our supplemental analysis suggested that only patient age and nasal LIPCOF were causatively related to preoperative OSDI and a decline in OSDI (Supplemental Table 2). Accordingly, symptomatic nasal CCh with a moderate or higher grade may be curable using only a surgical correction such as HFR-ES. In particular, younger patients with symptomatic CCh may have a stronger indication for surgery.

To date, 21 papers have introduced seven main types of surgical techniques for the treatment of CCh with or without DED (Supplemental Table 3). Of those, HFR-ES used in the current study generally has advantages of shorter operating time, better healing, avoidance of complications associated with sutures and scarring, and powerful cauterization without tissue charring^22,24,41^. Therefore, our HFR-ES for CCh could achieve a significant improvement of OS symptom and signs despite the short postoperative period.

There are some limitations to this study. First, although other potential contributors to CCh exist, we were unable to investigate them. For instance, blinking, lid tension, or lid wiper epitheliopathy play a role in tear spread and clearance, thereby affecting the OS environment chemically and physically. Additionally, the relationship between development of CCh and conjunctival fornixes, including the Kessing’s space (a tear reservoir), is unclear. Second, we did not inspect upper MGL grade and upper CCh, nor did we measure other objective indexes, including tear proteins or dynamics. Third, there was no control group for statistically analyzing baseline characteristics of enrolled patients. Lastly, the sample size and follow-up duration were limited. Accordingly, further large-scale studies with long-term observation should be conducted.

In conclusion, symptomatic CCh should be treated aggressively, since it is frequently accompanied by moderate to severe DED as well as by MGD. Although CCh is found in chronologically aged conjunctiva, it is caused by OS disruption and lid friction with tear instability, both of which result from DED and/or MGD. HFR-ES for CCh is an effective modality for reconstruction of the lower tear meniscus and reservoir. In terms of indications of HFR-ES for CCh, clinicians should consider the patient age and CCh severity in the nasal region. Furthermore, management for MGD should be maintained after CCh correction.

## Materials and methods

### Patients

This prospective study followed the tenets of the Declaration of Helsinki, and the prospective study protocol was approved by the Severance Hospital Institutional Review Board, Seoul, South Korea (IRB No. 4-2017-0362). We enrolled patients with CCh confirmed by slit-lamp biomicroscopy and AS-OCT, and ocular symptoms such as ocular discomfort, pain, foreign body sensation, and episodic tearing. Informed consent was obtained from all patients after explanation of the purpose and possible consequences of the study.

DED was diagnosed according to the Korean diagnostic criteria, as described elsewhere^42^. The study cohort included patients who fulfilled the following diagnostic criteria: OSDI score > 12; TBUT < 5 s, or CFS based on Oxford schema ≥ 1^3,43^. MGD was defined as (1) more than one lid margin abnormality, including irregularity, vascular engorgement, or anterior or posterior displacement of the mucocutaneous junction; (2) orifice obstruction such as plugging, pouting, or ridges; and (3) reduced meibum expression in response to moderate digital pressure^5,16,44^.

We excluded patients younger than 20 years, those who wore contact lenses, and those with any ocular history even in one eye, ocular surgery, ocular injury, ocular infection, allergy, or autoimmune disease, as well as patients using a punctual plug or topical ophthalmic medications. Patients who used artificial tears were instructed not to apply them for at least 12 h before an examination. Only the test values for the right eye were analyzed.

### Study protocol

After patients completed an OSDI questionnaire, clinical evaluations and automated measurements were performed in the following order to minimize the effect of the previous measurement on other measurements: MGL grade and LLT by LipiView II (TearScience Inc., Morrisville, NC); TMA at the nasal, central, and temporal regions of the lower lid margin by AS-OCT (RTVue, Optovue, Inc., Fremont, CA); LIPCOF score at the nasal, central, and temporal regions of the lower lid margin; CFS score; TBUT; and MGD staging. There was at least a 10-min interval between each test; all tests were performed in the same order. All clinical examinations were performed by one ophthalmologist (Y.W.J.), and the automated measurements were performed by another ophthalmologist (H.K.).

The OSDI questionnaire is a validated 12-item questionnaire that assesses symptoms of ocular irritation consistent with DED, their impact on vision-related function, and environmental triggers. It gives a range of 0 (no symptoms) to 100 (severe symptoms)^45^. LIPCOF (range, 0–3) was evaluated in the area perpendicular to the nasal, central, and temporal limbus on the bulbar conjunctiva above the lower lid (nasal LIPCOF, central LIPCOF, and temporal LIPCOF, respectively) with a slit-lamp microscope and classified using the optimized grading scale^8,10,37^. A further combined LIPCOF score (total LIPCOF; range, 0–9) was calculated by adding the nasal, central, and temporal LIPCOF. The CFS score (range, 0–5) was derived using the Oxford schema^43^, and tear film stability was determined by the TBUT using a sodium fluorescein strip (Haag-Streit, Koeniz, Switzerland). In terms of MGD staging, the examiner (Y.W.J) checked whether the following lid margin abnormalities were present: irregular lid margin, plugged MG orifices, vascular engorgement on the lid margin, and displacement of the mucocutaneous junction. Meibum expressibility was assessed on a scale of 0 to 3 in five glands in the lower lid, according to the number of glands expressible: 0, all five glands expressible; 1, three to four glands expressible; 2, one to two glands expressible; and 3, no gland expressible. Meibum quality was assessed in each of eight glands of the central third of the lower lid on a scale of 0 to 3 for each gland: 0, clear; 1, cloudy; 2, cloudy with granular debris; and 3, thick, like toothpaste. The scores of the eight glands were summed to obtain a total score (0 to 24): MGD stage 1, minimally altered expressibility (= 1) and secretion quality (≥ 2 and < 4); MGD stage 2, mildly altered expressibility (= 1) and secretion quality (≥ 4 and < 8); MGD stage 3, lid margin features of plugging, vascularity, and moderately altered expressibility (= 2) and secretion quality (≥ 8 and < 13); and MGD stage 4, lid margin features of displacement, and severely altered expressibility (= 3) and secretion quality (≥ 13)^46^.

LLT was measured using a LipiView interferometer, as described by Blackie et al.^47^ LLT is presented in interferometric color units (ICU), where 1 ICU corresponds to approximately 1 nm. The maximum LLT value is limited to be 100 nm. The MGs were imaged using an infrared camera system equipped with LipiView II. MGL grade was evaluated using a meiboscale of the lower lid: grade 0, no gland loss; grade 1, area of gland loss < 25% of the total gland area; grade 2, area of gland loss between 25 and 50%; grade 3, area of gland loss 50–75%; and grade 4, area of gland loss > 75%. TMA was measured using the AS-OCT based on the Fourier-domain technique (RTVue)^48^; AS-OCT was evaluated in the area perpendicular to the nasal, central, and temporal limbus on the bulbar conjunctiva above the lower lid (nasal TMA, central TMA, and temporal TMA, respectively).

### Surgical procedure of HFR-ES

HFR-ES was performed by a single experienced surgeon (K.Y.S.) using a fine-needle electrode (Fine Insulated Coated Needle, 004 Super Fine) and the 4.0-MHz radiowave system (Ellman Surgitron Dual Frequency; Ellman International, Inc., Hewlett, NY)^24,25^. Briefly, after instillation of 0.5% proparacaine (Alcaine; Alcon Laboratories, Fort Worth, TX), the extent of the CCh was determined, and a fine-needle electrode was inserted into the conjunctiva. The target conjunctiva was ablated while lifting to prevent the sclera from thermal burns or charring. Ablation was continued while retracting the electrode from the adjacent conjunctiva. All ablations were performed with the minimal power setting of 1 (of 100) in the coagulating mode, producing the least amount of lateral heat and tissue destruction. One to two seconds of ablation caused a gradual change in the conjunctiva to a dusky white color (blanching) (Fig. 1). Postoperatively, 0.5% levofloxacin (Cravit; Santen, Osaka, Japan) and 0.1% fluorometholone (Ocumetholone; Samil Pharmaceutical Co., Seoul, Korea) eye drops were initiated four times per day for 2 weeks.

### Statistical analysis

We used SPSS for Windows (version 21.0; IBM Corp., Armonk, NY) for statistical analyses. Normality of distribution was checked using the Kolmogorov–Smirnov test. The Wilcoxon signed-rank test was performed for comparison between pre- and post-operative values. Spearman’s correlation test confirmed the relationship between two values, with a correlation coefficient (*r*) of − 1 ≤ *r* ≤ 1. Stepwise multivariable linear regression analysis was applied to investigate the determinants of CCh severity or symptom relief following surgery, using criteria of probability-of-F-to-enter ≤ .05 and probability-of-F-to-remove ≥ .10. All regression model assumptions were evaluated by analyzing the condition index in the collinearity diagnostics to exclude the possibility of collinearity among the explanatory variables. The Durbin–Watson value was also investigated to confirm the independence of the residual errors. If the condition index was larger than 30, the last enrolled explanatory variable was excluded, and the other variables were included in the regression analysis. Only modes with a condition index larger than zero and less than 30 and a Durbin–Watson test statistic of around 2 were included. In all statistical tests, a *P*-value less than 0.05 was considered statistically significant^49–52^. Based on a priori power analyses using G * Power^53,54^ with preliminary results (n = 15; total predictors = 8 and tested predictors = 3), sample size of 40 achieved 85% power with a significance level (alpha) using a F test with post hoc analysis.

## Supplementary Information

Supplementary Tables.

## Abbreviations

CCh: Conjunctivochalasis
HFR-ES: High-frequency radiowave electrosurgery
DED: Dry eye disease
OSDI: Ocular surface disease index
CFS: Corneo-conjunctival fluorescein staining
TBUT: Tear break-up time
TMA: Tear meniscus cross-sectional area
LIPCOF: Lid parallel conjunctival folds
MGD: Meibomian gland dysfunction
MGL: Meibomian gland loss
LLT: Lipid layer thickness
AS-OCT: Anterior segment optical computed tomography
ATD: Aqueous tear deficiency
LTD: Lipid tear deficiency
