# Supplementary material for: The correction of conjunctivochalasis using high-frequency radiowave electrosurgery improves dry eye disease
Source: Sci Rep. 2021 Jan 28;11:2551. doi: 10.1038/s41598-021-82088-5 (PMC7844232; doi:10.1038/s41598-021-82088-5)
Supplement: Supplementary file 1 — Supplementary Tables. [file 41598_2021_82088_MOESM1_ESM.docx]

**The correction of conjunctivochalasis using high-frequency radiowave electrosurgery improves dry eye disease**

Yong Woo Ji (1,2), Hyojin Seong (1,2), Sujung Lee (3), Mutlaq Hamad Alotaibi (2,5), Tae-im Kim (2,4), Hyung Keun Lee (2,4), Kyoung Yul Seo (2)

1) Department of Ophthalmology, National Health Insurance Service Ilsan Hospital, Goyang, South Korea

2) Institute of Vision Research, Department of Ophthalmology, Yonsei University College of Medicine, Seoul, South Korea

3) Medical Library, National Health Insurance Service Ilsan Hospital, Goyang, South Korea

4) Corneal Dystrophy Research Institute, Department of Ophthalmology, Yonsei University College of Medicine, Seoul, South Korea

5) Department of Ophthalmology, Prince Mohammad Bin Abdulaziz Hospital, Riyadh, Saudi Arabia

None of the authors has any proprietary or financial interests regarding the materials and methods used in this study.

**Correspondence:**

Kyoung Yul Seo, MD, PhD

Department of Ophthalmology, Yonsei University College of Medicine

50-1 Yonsei-ro, Seodaemun-gu, Seoul 03722, Korea

E-mail: seoky@yuhs.ac

**Running head:** Effect of conjunctivochalasis correction on dry eye disease

**Address for reprints:** (same as Correspondence)

**Key words:** Conjunctivochalasis, dry eye disease, Meibomian gland dysfunction, lipid layer thickness, Tear film, LipiView

**Abbreviations:** Conjunctivochalasis, CCh; high-frequency radiowave electrosurgery, HFR-ES; dry eye disease, DED; Ocular Surface Disease Index, OSDI; corneo-conjunctival fluorescein staining, CFS; tear break-up time, TBUT; tear meniscus cross-sectional area, TMA; lid parallel conjunctival folds, LIPCOF; meibomian gland dysfunction, MGD; meibomian gland loss, MGL; lipid layer thickness, LLT; anterior segment optical computed tomography, AS-OCT; aqueous tear deficiency, ATD; lipid tear deficiency, LTD

**Supplemental Table 1.** Postoperative changes of clinical parameters in patients with dry eye disease after conjunctivochalasis correction.

|  | Preoperative |  | Postoperative | *P-*value |
| --- | --- | --- | --- | --- |
| **OSDI** | **42.65 ± 19.88** |  | **19.38 ± 4.34** | **< .001** |
| **TBUT, seconds** | **1.83 ± 0.55** |  | **3.75 ± 0.49** | **< .001** |
| **CFS score** | **1.43 ± 0.50** |  | **0.40 ± 0.50** | **< .001** |
| Nasal LIPCOF  Central LIPCOF  Temporal LIPCOF  Total LIPCOF | 2.28 ± 0.64  0.85 ± 0.98  2.10 ± 0.81  5.23 ± 1.82 |  | Completely  resolved |  |
| **Nasal TMA, mm^2^**  **Central TMA, mm^2^**  **Temporal TMA, mm^2^** | **0.0045 ± 0.0051**  **0.0103 ± 0.0086**  **0.0061 ± 0.0059** |  | **0.0289 ± 0.0325**  **0.0354 ± 0.0571**  **0.0214 ± 0.0094** | **< .001**  **< .001**  **< .001** |
| LLT, nm | 72.43 ± 27.44 |  | 75.53 ± 26.58 | .129 |
| MGD stage | 1.60 ± 1.53 |  | No change |  |
| MGL grade | 1.45 ± 1.36 |  | No change |  |

All data are shown as the mean ± standard deviation. *P-*value was obtained via Wilcoxon signed-rank test. Significant values are presented in bold type. OSDI, ocular surface disease index; TBUT, Tear break-up time; CFS, corneal and conjunctival fluorescein staining based on Oxford schema; LIPCOF, Lid-parallel conjunctival folds; TMA, tear meniscus cross-sectional area; LLT, Lipid layer thickness; MGD, Meibomian gland dysfunction; MGL, Meibomian gland loss.

**Supplemental Table 2.** Correlation and multivariable regression analyses evaluating the relationship of clinical parameters to preoperative ocular surface symptoms in dry eye patients with conjunctivochalasis**.**

| Dependent variable | Explanatory variables | Correlation analysis | |  | Multivariable analysis | | | | |
| --- | --- | --- | --- | --- | --- | --- | --- | --- | --- |
|  |  |  |  |  | Unstandardized coefficients | | Standardized coefficients | *P*-value | Adjusted R^2^ |
|  |  | *r* | *P*-value |  | B | SE | β |  |  |
| OSDI | **Age** | **- .359** | **.011** |  | **- .526** | **.195** | **- .328** | **.010** | **.429** |
|  | **LIPCOF**  **score – nasal** | **.500** | **.001** |  | **17.005** | **3.855** | **.547** | **< .001** |  |
|  | TMA – temporal | - .293 | .033 |  |  |  | - .198 | .126 |  |
|  | CFS score | .399 | .005 |  |  |  | .136 | .327 |  |

Significant values are presented in bold type. Each *P-*value was obtained via Spearman correlation analysis or stepwise multivariable regression analysis; *r*, Spearman’s correlation coefficient; SE, standard error; OSDI, ocular surface disease index; LIPCOF, Lid-parallel conjunctival folds; TMA, tear meniscus cross-sectional area; CFS, corneal and conjunctival fluorescein staining based on Oxford schema.

**Supplemental Table 3.** Various surgical techniques for correction of conjunctivochalasis in the literature.

| Surgical technique for CCh correction | Number of papers |  | Representative study | | | | | |
| --- | --- | --- | --- | --- | --- | --- | --- | --- |
|  |  |  | Author | Year | Enrolled patients | Follow-up | Outcomes |  |
| Conjunctival cauterization | 9^1-9^ |  | Çağlayan et al^9^ | 2018 | CCh and DED (n=20) | 1 mo | Significant reductions in OSDI Significant increases in TBUT and TMA |  |
| Conjunctival excision | 1^10^ |  | Petris et al^10^ | 2013 | Only CCh  (n=25) | 3 mo | Improvement of tearing symptom |  |
| Conjunctival excision with sutures | 3^11-13^ |  | Hara et al^11^ | 2011 | Only CCh  (n=15) | 3 mo | Improvement of symptoms  Significant increases in TBUT |  |
| Conjunctival excision with fibrin glue | 2^14,15^ |  | Acera et al^14^ | 2013 | Only CCh  (n=12) | 1 mo | Improvement of tearing symptom  Significant decrease in matrix metalloproteinase-9 of tears |  |
| Paste-pinch-cut conjunctivoplasty | 2^8,16^ |  | Doss et al^16^ | 2012 | Only CCh  (n=139) | 3 mo | Improvement of symptoms |  |
| HFR-ES | 2^17,18^ |  | Trivli et al^18^ | 2018 | CCh and DED (n=40) | 10 d | Improvement of symptoms |  |
| Laser conjunctivoplasty | 3^19-21^ |  | Yang et al^19^ | 2013 | Only CCh  (n=18) | 6 mo | Significant reductions in OSDI  Significant increase in TBUT |  |
| HFR-ES | Current study | | | | CCh and DED (n=40) | 1 mo | Significant reductions in OSDI and CFS  Significant increases in TBUT and TMA  Increase in LLT but not significant |  |

CCh, conjunctivochalasis; DED, dry eye disease; HFR-ES, high-frequency radiowave electrosurgery; Δ, difference between postoperative and preoperative values; OSDI, ocular surface disease index; TBUT, Tear break-up time; TMA, tear meniscus cross-sectional area; CFS, corneal and conjunctival fluorescein staining based on Oxford schema; LLT, Lipid layer thickness.

References

1. Haefliger, I. O., Vysniauskiene, I., Figueiredo, A. R. & Piffaretti, J. M. Superficial conjunctiva cauterization to reduce moderate conjunctivochalasis. *Klin Monbl Augenheilkd* **224,** 237-239 (2007).

2. Gumus, K., Crockett, C. H. & Pflugfelder, S. C. Anterior segment optical coherence tomography: a diagnostic instrument for conjunctivochalasis. *Am J Ophthalmol* **150,** 798-806 (2010).

3. Kashima, T., Akiyama, H., Miura, F. & Kishi, S. Improved subjective symptoms of conjunctivochalasis using bipolar diathermy method for conjunctival shrinkage. *Clin Ophthalmol* **5,** 1391-1396 (2011).

4. Nakasato, S., Uemoto, R. & Mizuki, N. Thermocautery for inferior conjunctivochalasis. *Cornea* **31,** 514-519 (2012).

5. Zhang, X. R., Zhang, Z. Y. & Hoffman, M. R. Electrocoagulative surgical procedure for treatment of conjunctivochalasis. *Int Surg* **97,** 90-93 (2012).

6. Chan, T. C. *et al.* Change in Tear Film Lipid Layer Thickness, Corneal Thickness, Volume and Topography after Superficial Cauterization for Conjunctivochalasis. *Sci Rep* **5,** 12239 (2015).

7. Arenas, E. and Munoz, D. A New Surgical Approach for the Treatment of Conjunctivochalasis: Reduction of the Conjunctival Fold with Bipolar Electrocautery Forceps. *ScientificWorldJournal* **2016,** 6589751 (2016).

8. Santiago, E. *et al.* Surgical techniques for the treatment of conjunctivochalasis: paste-pinch-cut conjunctivoplasty versus thermal cautery conjunctivoplasty. *Can J Ophthalmol* **52,** 308-312 (2017).

9. Caglayan, M., Kosekahya, P., Gurdal, C. & Sarac, O. Comparison of Electrocoagulation and Conventional Medical Drops for Treatment of Conjunctivochalasis: Short-Term Results. *Turk J Ophthalmol* **48,** 61-65 (2018).

10. Petris, C. K. and Holds, J. B. Medial conjunctival resection for tearing associated with conjunctivochalasis. *Ophthalmic Plast Reconstr Surg* **29,** 304-307 (2013).

11. Hara, S. *et al.* Evaluation of tear stability after surgery for conjunctivochalasis. *Optom Vis Sci* **88,** 1112-1118 (2011).

12. Yokoi, N., Komuro, A., Sugita, J., Nakamura, Y. & Kinoshita, S. Surgical reconstruction of the tear meniscus at the lower lid margin for treatment of conjunctivochalasis. *Adv Exp Med Biol* **506,** 1263-1268 (2002).

13. Yokoi, N. *et al.* Clinical impact of conjunctivochalasis on the ocular surface. *Cornea* **24,** S24-S31 (2005).

14. Acera, A., Vecino, E. & Duran, J. A. Tear MMP-9 levels as a marker of ocular surface inflammation in conjunctivochalasis. *Invest Ophthalmol Vis Sci* **54,** 8285-8291 (2013).

15. Brodbaker, E., Bahar, I. & Slomovic, A. R. Novel use of fibrin glue in the treatment of conjunctivochalasis. *Cornea* **27,** 950-952 (2008).

16. Doss, L. R., Doss, E. L. & Doss, R. P. Paste-pinch-cut conjunctivoplasty: subconjunctival fibrin sealant injection in the repair of conjunctivochalasis. *Cornea* **31,** 959-962 (2012).

17. Youm, D. J., Kim, J. M. & Choi, C. Y. Simple surgical approach with high-frequency radio-wave electrosurgery for conjunctivochalasis. *Ophthalmology* **117,** 2129-2133 (2010).

18. Trivli, A., Dalianis, G. & Terzidou, C. A Quick Surgical Treatment of Conjunctivochalasis Using Radiofrequencies. *Healthcare (Basel)* **6,** (2018).

19. Yang, H. S. and Choi, S. New approach for conjunctivochalasis using an argon green laser. *Cornea* **32,** 574-578 (2013).

20. Shin, K. H., Hwang, J. H. & Kwon, J. W. New approach for conjunctivochalasis with argon laser photocoagulation. *Can J Ophthalmol* **47,** 380-382 (2012).

21. Yang, J. *et al.* Near-infrared laser thermal conjunctivoplasty. *Sci Rep* **8,** 3863 (2018).
